# Modified Mediterranean-Ketogenic Diet and Carboxytherapy as Personalized Therapeutic Strategies in Lipedema: A Pilot Study

**DOI:** 10.3390/nu15163654

**Published:** 2023-08-20

**Authors:** Laura Di Renzo, Paola Gualtieri, Samanta Zomparelli, Gemma Lou De Santis, Silvia Seraceno, Claudia Zuena, Giulia Frank, Rossella Cianci, Domenico Centofanti, Antonino De Lorenzo

**Affiliations:** 1 Section of Clinical Nutrition and Nutrigenomics, Department of Biomedicine and Prevention, University of Rome Tor Vergata, Via Montpellier 1, 00133 Rome, Italy; 2 School of Specialization in Food Science, University of Tor Vergata, Via Montpellier 1, 00133 Rome, Italy; 3 PhD School of Applied Medical-Surgical Sciences, University of Rome Tor Vergata, Via Montpellier 1, 00133 Rome, Italy; 4 Department of Translational Medicine and Surgery, Fondazione Policlinico Universitario A. Gemelli, IRCCS, 00168 Rome, Italy; 5 Società Italiana di Medicina Estetica, 00195 Rome, Italy

**Keywords:** nutrition, ketogenic diet, carboxytherapy, SAT diseases, lipedema, body composition

## Abstract

In recent years, the use of the ketogenic diet as a proper nutritional treatment for lipedema has been hypothesized in the literature. This is the first clinical study evaluating the ketogenic diet and carboxytherapy in lipedema patients. In the present study, it was decided to use a modified Mediterranean ketogenic diet (MMKD) in combination with carboxytherapy. Since lipedema is characterized by microangiopathy, local hypoxia, and increased subcutaneous adipose tissue (SAT) deposition, carboxytherapy could improve painful symptoms and skin tone. A total of 22 subjects were included in the data analysis, divided into three groups; 8 patients underwent MMKD combined with carboxytherapy sessions (KDCB group), 8 underwent MMKD nutritional treatment alone (KD group), and 6 patients underwent only carboxytherapy sessions (CB group), for a total of 10 weeks of treatment for all three groups. It was observed that the ketogenic diet effectively induced weight and fat mass loss, including in the limbs, areas considered unresponsive to diet therapy in lipedema patients. However, the best results were obtained from the combination of the ketogenic diet and carboxytherapy, which showed improvements in both body composition and skin texture and a reduction in pain, along with an improvement in sleep quality. It would be helpful to conduct a clinical trial on a larger scale and over a more extended period to observe the results in the long term as well.

## 1. Introduction

Lipedema is an autosomal dominant inheritance, progressive, multifactorial, and disabling disease, characterized by an accumulation of pathological subcutaneous adipose tissue (SAT) in the superfascial area, microangiopathy, chronic tissue inflammation, and pain. Symptoms usually occur at puberty, pregnancy, or menopause, assuming the role of estrogens in the pathophysiological process. The diagnosis is clinical, the adipose tissue develops descending symmetrically and bilaterally, and the lower limbs are the most affected sites. It is classified into five types based on the distribution of the lipo-edematous adipose tissue and into four stages based on the severity of the disease [1]. The first clinical study on diet therapy in lipedema was published by our research group, and showed an improvement in overall body composition and in specifically affected areas (upper and lower limbs) with consumption of a low-carb diet and high-antioxidant-content foods, inspired by the Mediterranean diet [2]. In recent years, there has been a sharp increase in studies on the use of the ketogenic diet for various diseases, such as obesity, diabetes mellitus type II, Alzheimer’s disease, multiple sclerosis, and cancer [3,4]. It is characterized by an intake of carbohydrates <30 g/day, shifting from a glucose cellular metabolism to a lipid metabolism, thus inducing the oxidation of fats and ketosis (defined as a blood concentration of beta-hydroxybutyrate >0.5 mmol/L) [5,6]. In January 2021, a review was published encouraging the ketogenic diet in patients suffering from lipedema as the most effective therapy. Hypothesized mechanisms involved are a reduction in insulin and, therefore, in the lipogenesis and lipo-hypertrophy promoted by hyperinsulinemia; increased sense of satiety, both due to the intake of a greater quantity of lipids and by improving the cerebral sensitivity to leptin (which in subjects with an increase in adipose tissue can be reduced) [7]; modulation of inflammation; reduction in oxidative stress, carried out by beta-hydroxy-butyrate [8]; reduction in sensitivity to mechanical, thermal, and/or neuropathic pain after several weeks of ketosis [9]. Masino and Ruskin hypothesized that the restriction of carbohydrates reduces the neurons’ excitability, suppressing pain perception, reducing inflammation, and increasing the levels of adenosine, a natural analgesic [3]. Moreover, a decrease in appearance-related discomfort and depression following a reduction in weight and body fat, particularly in the most affected areas, was highlighted [10]. Additionally, several studies have shown that adopting a ketogenic regimen reduces depression, and improves attention, mood, and social interactions [11]. Recently, Cannataro et al. published a case report of a lipedema patient following a ketogenic diet as a nutritional intervention for two years, achieving significant weight loss, reducing pain, and, therefore, improving quality of life. However, to our knowledge, there are no clinical studies regarding the ketogenic diet in a group of lipedema patients.

Also, we wanted to evaluate the efficacy of carboxytherapy for a reduction in subcutaneous fat and a reduction in painful symptoms. Its use for therapeutic purposes dates back to the 1930s, when it was used as thermal therapy (baths in carbonated water) for treating patients with obliterating peripheral arterial disease [12]. The goal of carboxytherapy is to improve or restore circulatory function when damaged. This procedure is characterized by the administration of carbon dioxide (CO2) in the gaseous state by a subcutaneous or intradermal route through tiny needles (30 G) [13]. In addition, carboxytherapy stimulates microcirculation and increases the local blood flow, with a concomitant positive effect on fibroblastic activity and a lipolytic effect with an improvement in skin elasticity and skin irregularities caused by fat accumulation [14]. Since lipedema is characterized by microangiopathy, local hypoxia, and increased SAT deposition, carboxytherapy could improve painful symptoms and skin tone. Therefore, the primary outcome of this pilot study is to identify potential therapeutic strategies in a group of lipedema patients evaluating the efficacy of a personalized modified Mediterranean ketogenic diet (MMKD), with or without carboxytherapy, for a limited period on body composition parameters. The secondary outcome is to evaluate the effects of the treatments above on the state of health, pain perception, and quality of life.

## 2. Materials and Methods

### 2.1. Subjects and Study Design

**Figure 1:**
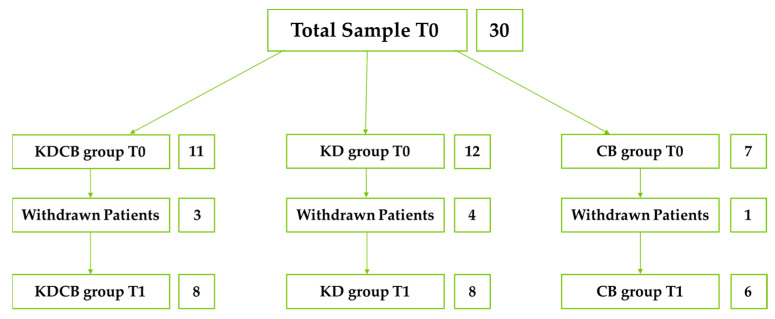
Flowchart.

Between May 2022 and May 2023, a pilot study was conducted at the Section of Clinical Nutrition and Nutrigenomics, University of Rome Tor Vergata, and 34 patients diagnosed with lipedema were enrolled. Inclusion criteria were diagnosis of lipedema, female sex, and consent to the protocol. Exclusion criteria were age <18 or >65 years; diagnosis of lymphedema; stage 4 lipedema; drug or alcohol abuse; psychotic disorders; acute or chronic renal failure; liver failure; neoplastic disease under treatment; pregnancy and lactation; menopause for more than 2 years; hepatitis B and C; HIV/AIDS; and COVID-19. Four patients declined to participate. Therefore, 30 patients were randomly divided into three groups; 11 patients underwent MMKD combined with carboxytherapy sessions (KDCB group), 12 underwent MMKD nutritional treatment alone (KD group), and 7 patients underwent only carboxytherapy sessions (CB group), for a total of 10 weeks of treatment for all three groups. From the KDCB group, 3 patients withdrew; from the KD group, 4 patients withdrew; from the CD group, 1 patient withdrew (major surgery, pregnancy, bereavement, COVID-19, relocation for work reasons). In the end, 22 patients completed the study, 8 in the KDCB group, 8 in the KD group, and 6 in the CB group (Figure 1).

A complete medical and nutritional evaluation was performed at baseline (T0) and after ten weeks (T1). All patients were interviewed via telephone every two weeks with a nutritional recall of the previous 24 h to monitor compliance with the dietary treatment and were questioned about any complications or side effects. Carboxytherapy sessions were performed once a week for the ten weeks of treatment by the same operator (S.S.).

All enrolled patients signed an informed consent form according to the principles of the Declaration of Helsinki. This trial is registered with ClinicalTrial.gov NCT01890070. Approval was obtained from the Ethics Committee of the Calabria Region Central Area Section (register protocol no. 146 17/05/2018).

### 2.2. Dietary Assessment and Intervention

At T0 and T1, a trained nutritionist assessed the subjects’ eating habits through a thorough food history. A 24 h recall and a food frequency questionnaire were conducted to identify the amount of intake and weekly frequency of different foods [15].

All subjects received the MMKD with a 20% calorie restriction of daily energy requirements. Daily protein intake was calculated as 2 g/kg of total lean mass (LM), according to Colica et al. [16]. The diet indicated for each day of the week the foods to be consumed, divided into five meals daily. The mean calorie distribution of the meals was as follows: 15%—breakfast, 10%—morning snack, 35%—lunch, 10%—afternoon snack, and 30%—dinner. The daily macronutrient intake of the diet was divided as follows: <10% of total kcal/day of carbohydrates (<30 g day), 15 g of fiber, 20–25% of total kcal/day of protein (of which 20% was of vegetable origin), and 70% of total kcal/day of lipids (of which 48% was of polyunsaturated fatty acids (PUFA) and monounsaturated fatty acids (MUFA)). The bromatological composition of the diet was obtained using Dietosystem^®^ dietary analysis software (version 12.00.13, DS Medica SRL, Milan, Italy).

The main characteristics of the MMKD are as follows: seasonal vegetables; foods rich in mono- and polyunsaturated fats, such as olive oil, nuts, avocado, oily fish, and fresh cheeses with reduced saturated fat content; lean meat of organic origin; and use of herbs and spices to reduce the amount of added salt. Processed and preserved foods such as frozen ready meals, cured meats, canned products and sausages, simple carbohydrates and sugars, sugary alcoholic and soft drinks, and fresh fruits (except for red fruits) were excluded.

The Mediterranean adequacy index (MAI) was calculated using the ratio of caloric intake (% kcal/day) from typical Mediterranean carbohydrates and foods (such as fruits, vegetables, bread, pasta, extra virgin olive oil, fish, and wine) to nontypical foods (such as milk and dairy products, meat, eggs, sweets, sugar, and alcoholic beverages). MAI values are considered acceptable when the value is >5 and 100% adequate if >15 [17].

### 2.3. Carboxytherapy

The method consists of the supply of CO2 through highly sophisticated electronic machinery (CarbomedCO2, Physioled, Taumed, Rome, Italy), which allows supplying of pure gas, free from contaminants, with specific preset parameters of dose, flow, speed, and temperature. The CO2 passes through a disposable sterile tube at the end of which a 30-gauge 13 mm needle or 4 mm microlance is inserted. The dose and speed depend on the treatment site and the clinical indication. The injection can be superficial, intradermal, or subcutaneous. The rate that CO2 takes to cross the tissue varies according to the characteristics of the tissue of the subject being treated [18]. In our study, CO2 was delivered subcutaneously via a 30-gauge needle angled at 45° to the tissue to be treated at a temperature of 43 °C. Up to 500 mL per side per session can be administered. A total of 2400 mL per session was delivered, with an average flow of 80 mL/min. The protocol included ten sessions, one time a week. Carboxytherapy was performed on the lower limbs bilaterally in the following areas: trochanteric, midthigh anterior, inner thigh, posterior thigh, and popliteal.

CO2 determines increased local blood flow rate; increased arteriolar and metarteriolar sphygmicity (short-term effect); stimulation of true and false neoangiogenesis, with an increase in microcirculation (long-term effect); extero-receptor activation, linked to hyper-distension of subcutaneous tissues, with the production of bradykinin, increase in cyclic adenosine monophosphate (cAMP), and activation of intra-adipocyte lipase; and Bohr effect amplification, with the more significant release of oxygen (O2) at the tissue level [19]. Possible adverse effects are local pain associated with crepitus or burning sensation and possible bruising. Serious adverse events are rare and were not detected during our protocol. It cannot cause embolism, and it is not toxic [20].

### 2.4. Anthropometrics, Body Composition, and Basal Metabolism

After a 12 h overnight fast, an anthropometric assessment was performed for each patient. Body weight and height were measured with a scale and stadiometer (Invernizzi, Rome, Italy) while the subject was standing wearing underwear. Data were collected to the nearest 0.1 kg and 0.1 cm, respectively. Body mass index (BMI) was calculated as body weight (kg)/height (m^2^) and classified according to the World Health Organization (WHO) [21]. Hip and waist circumferences were measured with a flexible, non-stretchable metric tape.

Body composition was assessed according to the standard method [22]. Patients were asked to remove all clothing (except underwear), shoes, and any metal objects. Whole and segmental fat mass (FM) (kg) were assessed using dual X-ray absorptiometry (DXA) (Primus, X-ray densitometer; software version 1.2.2, Osteosys Co., Ltd., Guro-gu, Seoul, Republic of Korea). The effective radiation dose for this procedure is about 0.01 mSv. The intra- and intersubject coefficient of variation (CV% = 100 SD/mean) ranged from 1 to 5%. The coefficients for this instrument for five participants undergoing six scans over 9 months were 2.2% for FM and 1.1% for fat-free mass (FFM) and LM. Total FM (% FM) was calculated as total body FM (total FM) divided by the total mass of all tissues, including total body bone (TBBone), as follows: % FM = [total FM/(total FM + total LM + TBBone)] × 100.

Based on % FM, subjects are classified as normal weight (NW) for % FM < 30, and obese for % FM ≥ 30. Android and gynoid distribution were confirmed when gynoid and android total fat tissue (%) were >30 [23,24].

The appendicular skeletal muscle mass index (ASMMI) was calculated as follows:

(LM legs + LM arms)/height (m^2^). The intermuscular adipose tissue (IMAT) was calculated according to Colica et al. [16] with the following formulas: Log (IMAT) = −2.21 + (0.12 × fat) + (−0.0013 × fat^2^) for women.

Basal metabolic rate (BMR) was calculated using the De Lorenzo formula [25]:

BMR = [(3941 × VO2) + (1106 × VCO2)] × 1.44

VO2 = LM kg (DXA) × 5.3 (f)

VCO2 = VO2 × 0.85

### 2.5. EUROQOL-5 Dimension (EQ-5D)

This test was constructed to become a generic measure of quality of life, and to be short, easy to use, and self-administered. It was validated through collaboration with researchers from Northern Europe (Finland, the Netherlands, UK, Sweden), who have worked on the European Quality of Life project since 1990. It is a standardized instrument that can be used for different purposes; in this case, it was used to assess the possible changes in health status at other times (T0 and T1) and to contribute to the standardized assessment of the verification of the effectiveness of the dietary treatment used in patients with lipedema.

The second section of the EQ-5D includes an assessment using visual analog scale (VAS) graphically represented by a graded scale ranging from 0 (the worst possible health status) to 100 (the best possible health status) on which the respondent indicates the level of perceived health status. In this case, the EQ-5D evaluates the comparison of the change in the numbers obtained between the first part and the second part at T0 and T1, particularly in assessing any improvements regarding not only general health status but also pain, discomfort, and the psychological aspect related to depression and anxiety [26].

The EQ-5D is reported in Appendix A.

### 2.6. Fibromyalgia Tests

#### 2.6.1. Fibromyalgia Assessment Status (FAS)

Examinations of patients with lipedema showed that the areas of increased pain and soreness overlapped with those of fibromyalgia, even though this condition does not characterize lipedema. Therefore, to assess the effect of diet and carboxytherapy on pain and asthenia, it was decided that the FAS would be used. This simple self-administered test assesses asthenia, pain, and sleep disturbances based on the 16 non-joint sites listed on the self-assessment pain scale (SAPS) in a single measure (range: 0 to 10) [27].

The FAS is reported in Appendix A.

#### 2.6.2. Fibromyalgia Severity Scale (FSS)

This is an instrument used to assess symptoms and signs related to pain in fibromyalgia pathology. It consists of the diffuse pain index, which refers to the distribution of the pain symptom in the last week at the time of observation; generalized pain index, which details the body regions affected by pain; and symptom severity index, which considers symptoms such as asthenia, nonrestorative sleep, and cognitive problems, reported in the last week, and symptoms such as migraine, abdominal pain or cramps, and depression, reported in the last six months [27].

The FSS is reported in Appendix A.

#### 2.6.3. Revised Fibromyalgia Impact Questionnaire (FIQR)

The FIQR is an updated version of the fibromyalgia impact questionnaire (FIQ). It consists of twenty-one numerical rating scales from 0 to 10 (where 10 is “worst”) that explore the three main domains of function, widespread impact, and symptoms. The revised version expanded the original scale by adding new questions about sensitivity, balance, memory, and environmental sensitivity. All questions refer to the previous seven days. The final score (range 0–100, with higher values indicating greater severity of illness) is calculated as follows: the algebraic sum of the 9-item function domain (range 0–90) is divided by three; the algebraic sum of the 2-item general impact domain (range 0–20) remains unchanged; and the algebraic sum of the 10-item symptom domain (range 0–100) is divided by two. These three partial scores are then added together [28].

The FIQR is reported in Appendix A.

### 2.7. Difficulties in Emotional Regulation Scale (DERS)

The DERS is a self-reported measure that aims to assess emotion dysregulation. It is widely used in treatment and research settings for adults with emotional disorders (e.g., bipolar, anxiety, depression, obsessive-compulsive disorder, trauma, and stress-related disorders), so it is useful for investigating the possible presence of psychological disorders in patients with lipedema.

The model on which DERS is based proposes six emotion regulation factors, translated into six subscales: (a) lack of awareness and understanding of emotions (“I am mindful of my feelings”, with reverse scoring); (b) lack of acceptance of emotions (“I have difficulty making sense of my feelings”); (c) difficulty controlling impulses (“When I am angry, I get out of control”); (d) difficulty engaging in goal-oriented cognitions and behaviors when distressed (goals; “When I’m angry, I have a hard time getting the job done”); (e) unwillingness to accept specific emotional responses (“When I’m angry, I get angry at myself for feeling that way”); and (f) lack of access to strategies for feeling better when distressed (“When I’m angry, I think there’s nothing I can do to feel better”). Developed initially as a 36-question measure, a short 18-question form was created that retains the original six subscales. The measure is scored so that higher scores reflect more significant impairment or dysregulation [29,30,31].

The DERS is reported in Appendix A.

### 2.8. Yale Food Addiction Scale 2.0 (YFAS 2.0)

Lipedema patients may present with eating disorders. Therefore, a validated diagnostic tool for food addiction (FA), the YFAS, was used to identify individuals with addictive eating behaviors. The original YFAS (25 questions) was based on the Diagnostic and Statistical Manual of Mental Disorders (DSM) IV for substance dependence, including the consumption of highly palatable foods (e.g., pizza, ice cream, and chocolate). Later, the scale was replaced with the YFAS 2.0 (35 questions) in response to the revised criteria for substance-related disorders and addictions in the DSM-5. YFAS 2.0 introduced the following diagnostic criteria: use despite interpersonal or social consequences and, in physically dangerous situations, craving. A severity classification was also introduced.

The YFAS version 2.0 is a thirty-five-question questionnaire with an eight-point response scale. It measures the eleven criteria of substance-related disorders and addictions to food and eating. For the potential “diagnosis” of food addiction, at least two of the eleven addiction and substance use disorder criteria must be met, and significant impairment must be present. The YFAS also measures eating behavior impairment.

Both versions have two scoring options: a symptom count (YFAS range = 0–7, YFAS 2.0 range = 0–11), a diagnostic score that predicts the achievement of a symptom threshold (YFAS ≥ 3, YFAS 2.0 ≥ 2), and the presence of clinically significant impairment or distress (self-perceived). With respect to the first version, the YFAS 2.0 differentiates the severity level of FA based on symptom count (mild 2–3, moderate 4–5, severe ≥ 6), paralleling the DSM-5 severity [32,33].

The YFAS 2.0 is reported in Appendix A.

### 2.9. Statistical Analysis

The data collected before statistical evaluations were analyzed for the presence of outliers, and the Shapiro–Wilk test was performed to evaluate variable distribution. The data presented are expressed as mean and standard deviation, to evaluate differences between the times. Related sample t-tests or Wilcoxon signed rank tests were used to assess the differences in the variables examined at the follow-up. Results were significant for *p*-value < 0.05. Statistical analysis was performed using IBM SPSS Statistics V25.0 (SPSS, Chicago, IL, USA).

## 3. Results

### 3.1. Dietary Components

At T0 and T1, the results of dietary component analysis (macro- and micronutrients and nutritional indices) were calculated using food frequency. The average dietary components of the patients’ eating habits at T0 and the average dietary components of KD at T1 were compared (Table 1). Significant differences are shown for the following parameters: decreased carbohydrates (%) (*p* = 0.001), reduced sugars (%) (*p* = 0.001), decreased fiber (g) (*p* = 0.011), and increased animal proteins (%) (*p* = 0.001). In addition, statistically significant results were obtained for increased MUFA and PUFA content (*p* = 0.001). Statistically significant increases in vitamin D (μcg) (*p* = 0.001) and vitamin E (mg) (*p* = 0.001) content were also observed, while vitamin C (mg) was significantly reduced (*p* = 0.07). A significant increase in the MAI when comparing the dietary patterns followed at T0 and T1 (*p* = 0.001) was observed.

### 3.2. Sample Description, Anthropometrics, and Body Composition

After screening through the inclusion criteria, 30 subjects, all female, were included in the data analysis (Table 2).

According to BMI and % FM, 13.8% of patients were found to be normal weight, 20.7% normal weight obese, and 65.5% obese. According to the distribution of FM, 44.8% had a gynoid distribution, 55.2% had a mixed-type distribution, and none had an android distribution. An ANOVA test was performed to analyze the BMI and % FM differences between the three groups at T0. No differences were highlighted (respectively *p* = 0.63 and *p* = 0.83).

The percentage of different stages of lipedema identified in the sample was as follows: 20% of patients were stage 1, 60% were stage 2, 20% were stage 3, and none were stage 4.

Then, the sample was divided into three groups: 11 patients treated with the ketogenic diet and carboxytherapy (KDCB group), 12 with the ketogenic diet only (KD group), and 7 with carboxytherapy only (CB group).

After 10 weeks of treatment, a significant decrease in most of the parameters analyzed was shown (Table 3). Weight and BMI were significantly reduced in both the KDCB (*p* = 0.009, *p* = 0.008) and the KD groups (*p* = 0.003, *p* = 0.008). These parameters were not significantly different in the CB group. In relation to circumferences, only the hip was significantly different in both the KDCB and KD groups (*p* = 0.02, *p* = 0.0001), while waist circumference was significantly reduced only in the KD group (*p* = 0.03). No difference in the CB group was detected.

Regarding DXA parameters, in the KDCB group, total FM (expressed in grams and percentage) was significantly reduced (*p* = 0.005, *p* = 0.012), as was leg FM, in grams and percentage (*p* = 0.001, *p* = 0.02). The same results were obtained in the KD group for total FM g and % (*p* = 0.019 and *p* = 0.044), while FM in legs was significantly reduced only in g (*p* = 0.015). Differently, in the CB group, no significant results related to these parameters were found. In addition, significant decreases in the percentage of android and gynoid FM and IMAT were shown in both the KDCB group (*p* = 0.0011, *p* = 0.003, *p* = 0.026) and the KD group (*p* = 0.029, *p* = 0.03, *p* = 0.007). In contrast, in the CB group, none of these parameters changed significantly (Table 4).

The total LM obtained from DXA analysis was also found to be statistically unaffected in all three groups. Also, the ASMMI was statistically unaffected in all three groups. Evaluation of LM in legs for the KDCB, KD, and CB groups confirmed the unaltered statistical trend associated with total LM.

In all three groups, for the other DXA parameters, after ten weeks no statistical differences were observed.

**Figure 2:**
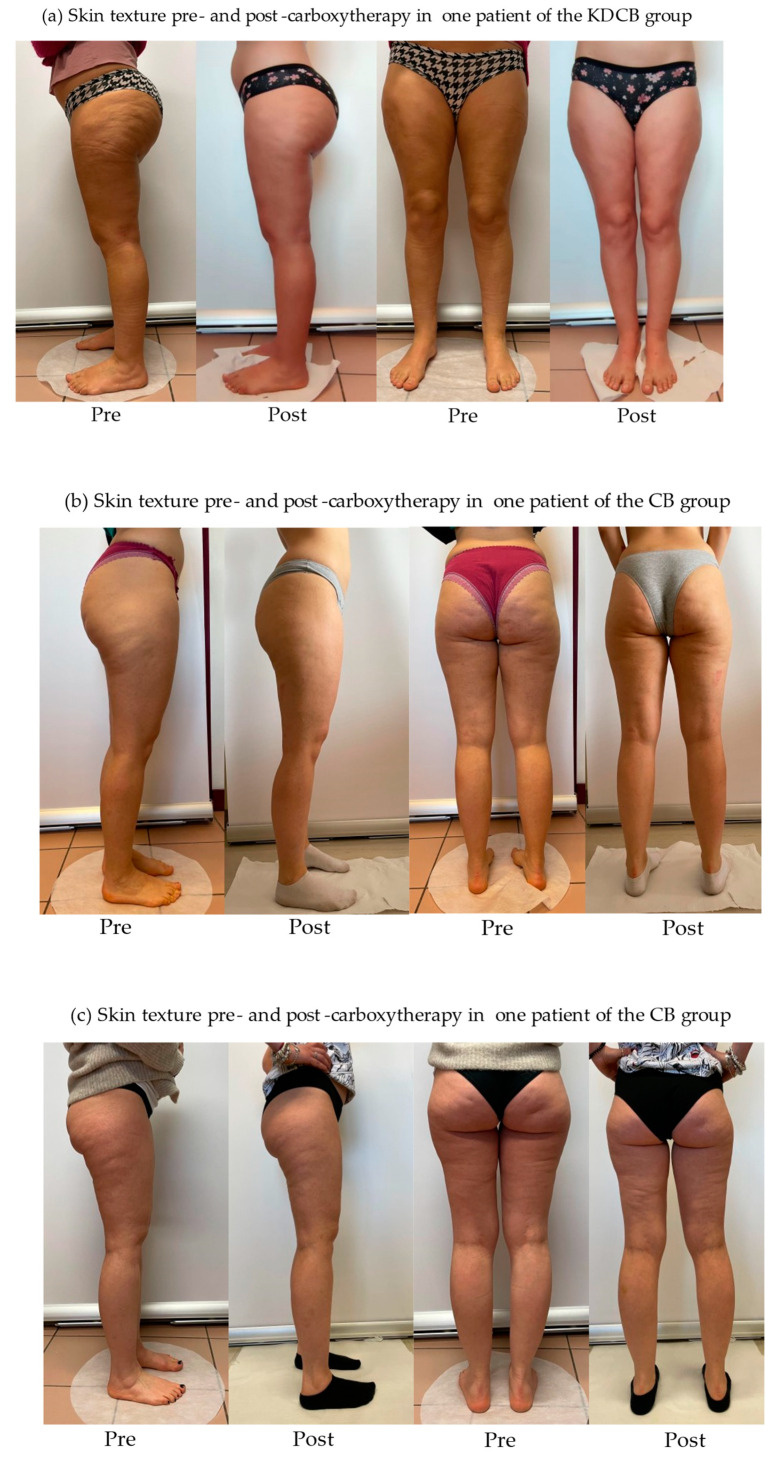
Skin texture pre- and post-carboxytherapy.

In the KDCB and CB groups, an improvement in skin texture in the legs was observed, as shown in the pictures (Figure 2a–c).

### 3.3. Quality of Life, Fibromyalgia, and Psychometric Tests

Different tests on quality of life and pain were administered to the three groups at baseline and follow-up. A statistically significant improvement was found in the KDCB group at the level of the FAS, which investigates asthenia, pain, and sleep disturbance (*p* = 0.049), and of the FIQR, which explores the three main domains of symptoms, function, and general impact (*p* = 0.032), indicating significant improvements in sleep quality and quality of life and a reduction in pain after 10 weeks of treatment.

No other significant changes were found in the other test scores and in the other groups (Table 5).

## 4. Discussion

Conservative therapeutic strategies in lipedema aim to reduce symptoms and prevent complications and progression of the disease [34]. Thus, to improve the quality of life of patients with lipedema, it is essential to find a dietary strategy aimed at weight loss and FM reduction in the typical areas of lipedema, such as the lower limbs, but also aimed at reducing pain caused by the orthostatic edema and the expansion of inflamed subcutaneous tissue. There seems to be a widespread misconception that limb volume and symptoms of lipedema do not respond to weight loss [1,35]. In fact, this concept has recently been challenged as contrary to the clinical experience of other scholars [2]. Regardless of BMI, early nutritional therapy is recommended at the time of diagnosis to prevent the development of obesity and the progression of lipedema [36].

The nutritional treatment, in this case, is a diet inspired by the Mediterranean diet in terms of food choice but ketogenic given that it is low in carbohydrate intake, low in salt and simple sugars, includes no processed foods, and is high in antioxidant foods, such as foods with high concentrations of PUFA and MUFA (e.g., oily fish, nuts, and extra virgin olive oil). These nutrients play an essential role in the inflammatory process by promoting an anti-inflammatory effect [37]. In fact, the values of some vitamins typical of the Mediterranean diet involved in antioxidative processes, such as vitamin D and vitamin E, appear to be increased (*p* = 0.001). Moreover, our results showed an improvement in the MAI between the diet followed by the patients before our dietary intervention and the diet followed according to our MMKD pattern, although it did not reach the level of Mediterranean adequacy (the KD being devoid of complex carbohydrates, fruits, and legumes), as expected given the low amount of carbohydrates. This goes to underscore how the MMKD used was rich in antioxidant and health-beneficial foods [38,39]. However, the observed significant reduction in vitamin C could be related to the lack of foods such as fresh fruit.

In fact, it has already been seen that a low-calorie diet based on foods rich in anti-inflammatory and antioxidant nutrients could contribute to the well-being of patients with lipedema [2]. In our previous study, after four weeks of treatment, an unexpected significant loss of FM in the legs and arms was detected, resulting in the first effective nutritional treatment for lipedema. A second important result was the improvement in quality of life, with reductions in asthenia, pain, and anxiety.

In recent years, the use of the ketogenic diet as a valid nutritional treatment for lipedema has been hypothesized in the literature [40]. In fact, it has an interesting rational application in this disease, both for the reduction in insulinemia (and thus hyperinsulinemia-promoted adipogenesis) and for an anti-inflammatory effect [7]. However, at present, there is a lack of clinical trials in the literature on the evaluation of the efficacy of the ketogenic diet in patients with lipedema.

In the present study, it was decided to use an MMKD, for the reasons discussed above, in combination with carboxytherapy. Indeed, lipedema is characterized by microangiopathy and local hypoxia, so we hypothesized that carboxytherapy, through the restoration of microcirculation, could improve painful symptoms and skin tone. Brandi et al. studied the effect of CO2 therapy on skin laxity in the knee and thigh region, observing an improvement in skin texture, as also expected for lipedema [14]. In addition, Pianez et al. [41] evaluated the efficacy of carboxytherapy in reducing cellulite in the buttocks and thigh area, noting an improvement due to the remodeling of collagen fibers and an increase in their quantity. A local lipolytic effect was also observed.

However, data on carboxytherapy are quite divergent in the literature, and many authors do not mention the parameters used regarding volume, infusion rate, and application technique, thus making it difficult to suggest a more effective procedure.

In any case, to the best of our knowledge, this is the first clinical study evaluating the effect of carboxytherapy on women with lipedema. What was found in our study was a significant improvement in skin texture, as shown (Figure 2a–c).

In contrast to the previous literature [1,35,42,43], which indicates lipedema fat as resistant to weight loss, our results show statistically significant losses of body weight, waist and hip circumferences, and total FM (g, %), and a reduction in IMAT, but especially a loss of lower limb (g) and gynoid (%) FM in both the KDCB and KD groups (*p* = 0.001, *p* = 0.015 and *p* = 0.003, *p* = 0.03, respectively). These results confirm and strengthen the evidence on diet therapy from our previous study [2].

Furthermore, contrary to what was observed for very low-calorie ketogenic diets (VLCKD) [16], LM was maintained throughout the duration of the study. This was also confirmed by the sarcopenia index ASMMI, which did not change in all groups (*p* = 0.585, *p* = 0.384, *p* = 0.538).

The same results for FM were not found in the group that performed only carboxytherapy. In fact, the primary goal of carboxytherapy is to restore and improve skin microcirculation, not to reduce fat mass; any lipolysis is only a secondary effect [44,45]. Other results to note are the significant improvements in sleep quality, asthenia, and pain reduction in the KDCB group (*p* = 0.049, *p* = 0.032). These data could reflect a reduction in inflammatory phenomena induced by adipose tissue as a consequence of a customized MMKD rich in antioxidant nutrients [46], in combination with the synergistic effect of carboxytherapy, which would induce an improvement in microcirculation and thus pain.

Moreover, a relationship between vitamin D levels and the development of anxiety and depression was previously observed [47]. The increased vitamin D content compared with baseline may have contributed to improving symptoms (pain), quality of sleep, and daily life functions in general in our patients.

The main limitations of this study are the small number of participants and the short intervention period. It will be necessary to further investigate the efficacy of the proposed treatments on a larger sample and for longer periods of time. In addition, this study lacks a comparison with a sample of non-lipedema patients to compare the results obtained. Moreover, it might be useful to assess patients’ oxidative and inflammatory status to check the antioxidative and anti-inflammatory power of nutrients administered through the diet.

## 5. Conclusions

To our knowledge, this is the first clinical study evaluating the ketogenic diet and carboxytherapy in lipedema patients.

It was observed that the ketogenic diet effectively induced weight and fat mass loss, including in the limbs, areas considered unresponsive to diet therapy in lipedema patients. The best results were obtained from the combination of the ketogenic diet and carboxytherapy, which showed both an improvement in body composition and a reduction in pain, along with an improvement in sleep quality.

This study confirms the need, according to the consensus of the various international guidelines, to maintain a multidisciplinary and integrated approach to lipedema, demonstrating that no isolated treatment, at present, can be completely effective.

Nutrition should have a primary role in the management of lipedema, as it could be, in the long term, an effective tool to reduce the low-grade inflammation that is almost always present in this condition. In parallel, it is necessary to continue studying this disease and its pathophysiology to have targeted therapeutic strategies.

Given the promising results of this pilot study, it would be useful to conduct a clinical trial over a more extended period and on a larger scale to observe the results in the long term as well.

## Figures and Tables

**Table 1:** Average dietary components of the patients’ eating habits at T0 and average dietary components of the KD at T1.

	Media T0	Media T1	*p*-Value
Energy (Kcal)	1551.69 [329.96]	1512.5 [140.73]	0.617
Proteins (% Kcal)	22.88 [7.12]	22.81 [0.7]	0.969
Vegetable proteins (% Kcal)	22.52 [10.17]	20.08 [2.12]	0.373
Animal proteins (% Kcal)	65.96 [9.93]	77.95 [2.07]	<0.001 *
Carbohydrates (% Kcal)	28.9 [11.85]	5.95 [0.67]	<0.001 *
Sugars (% Kcal)	22.68 [13.79]	3.5 [0.26]	<0.001 *
Fiber (g)	18.32 [5.97]	14.17 [0.9]	0.011 *
Lipids (% Kcal)	47.84 [10.36]	71.23 [0.93]	<0.001 *
SFA (% Kcal)	14.92% [9.93%]	15.70% [0.82%]	0.759
PUFA (% Kcal)	5.10% [1.93%]	8.55% [0.51%]	<0.001 *
MUFA (% Kcal)	22.80% [4.96%]	40.60% [0.70%]	<0.001 *
Vit C (mg)	154.41 [72.32]	101.34 [10.21]	0.007 *
Vit D (μcg)	2.42 [1.89]	4.91 [0.68]	<0.001 *
Vit E (mg)	14.94 [5.75]	22.87 [1.81]	<0.001 *
MAI	0.87 [0.65]	2.63 [0.75]	<0.001 *

SFA, saturated fatty acid; PUFA, polyunsaturated fatty acid; MUFA, monounsaturated fatty acid; Vit, vitamin; MAI, Mediterranean adequacy index; KDCB, ketogenic diet and carboxytherapy group; KD, ketogenic diet group. Standard deviation in square brackets. Statistical significance was attributed as * *p* < 0.05.

**Table 2:** Description of total sample at T0.

Sample T0	
Total (n)	30
Sex (M/F)	30 (F)
Age (years)	46 [7.4]
Height (cm)	160.65 [6.2]
Weight (kg)	73.8 [17]
BMI (kg/m^2^)	28.6 [6.5]

Standard deviation in square brackets.

**Table 3:** Comparison of anthropometrics at T0 and at T1 in the 3 treatment groups.

	KDCB T0	KDCB T1	*p*-Value	KD T0	KD T1	*p*-Value	CB T0	CB T1	*p*-Value
Weight (Kg)	72.64 [21.17]	69.29 [21.5]	0.009 *	74.26 [11.53]	70.73 [11.55]	0.003 *	76.78 [24.88]	76.07 [24.78]	0.168
BMI (Kg/m^2^)	28.33 [7.3]	27.02 [7.51]	0.008 *	27.18 [4.66]	25.88 [4.39]	0.006 *	30.68 [10.93]	30.4 [10.92]	0.211
Waist Circumference (cm)	80.5 [16.06]	77.38 [15.26]	0.069	82.25 [10.94]	79.19 [10.52]	0.03 *	83.37 [17.03]	83.02 [16.57]	0.527
Hip Circumference (cm)	107.41 [13.4]	104.94 [13.04]	0.02 *	109.66 [7.87]	105.19 [8.54]	0.0001 *	112.32 [20.8]	113.55 [19.93]	0.239

KDCB, ketogenic diet and carboxytherapy group; KD, ketogenic diet group; CB, carboxytherapy group; T0, baseline; T1, after 10 weeks. Standard deviation in square brackets. Statistical significance was attributed as * *p* < 0.05.

**Table 4:** Comparison of body composition parameters at T0 and T1 in the 3 treatment groups.

DXA Parameters	KDCB T0	KDCB T1	*p*-Value	KD T0	KD T1	*p*-Value	CB T0	CB T1	*p*-Value
Total fat tissue (%)	36.39 [9.21]	34.38 [9.6]	0.012 *	38.03 [7.69]	36.35 [9.83]	0.044 *	38.78 [9.52]	37.42 [10.43]	0.153
Total fat tissue (g)	26,825.88 [15,049.11]	24,619.63 [15,214.81]	0.005 *	29,135.88 [9149.12]	26,489.17 [11,013.63]	0.019 *	32,070.5 [18,361.24]	29,948.5 [17,215.04]	0.064
Total leg fat tissue (%)	42.44 [8.45]	40.49 [8.93]	0.020 *	46.08 [6.02]	44.97 [8.03]	0.075	45.63 [6.37]	45.27 [5.98]	0.598
Total leg fat tissue (g)	13,008.63 [5692.33]	11,702.88 [5722.94]	0.001 *	14,616.63 [4120.1]	13,264.5 [4021.63]	0.015 *	14,243.5 [6219.5]	14,108.33 [6473.1]	0.673
Total arm fat tissue (g)	2177.25 [682.81]	2266 [955.18]	0.676	2336.38 [884.28]	2167.5 [959.96]	0.664	2557.67 [1551.81]	2674.67 [1820.62]	0.564
Android total fat tissue (%)	37.63 [12.71]	33.75 [13.56]	0.0011 *	41.44 [10.09]	35.47 [17.34]	0.029 *	41.67 [11.91]	40.97 [12.45]	0.666
Gynoid total fat tissue (%)	43.95 [7.54]	40.91 [7.91]	0.003 *	46.66 [5.92]	43.27 [8.63]	0.030 *	47.55 [6.4]	46.48 [6.55]	0.314
Total lean mass (g)	41,295.75 [10,954.4]	40,645.25 [10,826.55]	0.243	43,512.38 [4267.88]	41,546 [5058.08]	0.139	43,379 [7137.34]	42,949.67 [6532.6]	0.530
Total leg lean mass (g)	15,772.88 [3054.57]	15,197.63 [3291.54]	0.114	15,725 [2284.37]	14,888.33 [1940.51]	0.397	15,160.55 [3177.59]	15,301.67 [3907.32]	0.815
Total arm lean mass (g)	3488.5 [596.14]	3833.5 [398.06]	0.121	3509 [723.16]	3634.17 [333.77]	0.426	3580 [700.9]	3921.17 [831.92]	0.346
IMAT	0.94 [0.45]	0.83 [0.45]	0.026 *	1.17 [0.43]	1.05 [0.48]	0.007 *	1.16 [0.61]	1.1 [0.62]	0.111
ASMMI	7.54 [0.87]	7.45 [1.08]	0.585	7.03 [0.8]	6.63 [0.41]	0.384	7.42 [1.5]	7.62 [1.7]	0.538

KDCB, ketogenic diet and carboxytherapy group; KD, ketogenic diet group; CB, carboxytherapy group; IMAT, intramuscular adipose tissue; ASMMI, appendicular skeletal muscle mass index; T0, baseline; T1, after 10 weeks. Standard deviation in square brackets. Statistical significance was attributed as * *p* < 0.05.

**Table 5:** Comparison of test parameters in the three groups.

	KDCB T0	KDCB T1	*p*-Value	KD T0	KD T1	*p*-Value	CB T0	CB T1	*p*-Value
EuroQ_tot	45.14 [11.98]	35 [16.41]	0.141	63.63 [38.65]	75.71 [31.42]	0.729	46.83 [33.2]	56.67 [27.51]	0.067
FAS	12.51 [5.88]	8.63 [6.06]	0.049 *	16.88 [6.71]	16.44 [6.32]	0.860	10.9 [7.25]	15.2 [2.11]	0.155
FIQR_tot	41.43 [24.52]	31.33 [21.42]	0.032 *	66.5 [42.14]	64.52 [40.32]	0.730	51.06 [36.26]	66.39 [23.59]	0.138
FSS	12.29 [8.42]	9.63 [7.82]	0.119	16.38 [8.6]	16 [5.86]	0.643	12.17 [9.41]	15.83 [8.13]	0.175
DERS tot	81.14 [22.45]	76.63 [24.77]	0.408	84.38 [29.13]	79.71 [25.34]	0.250	70.67 [16.27]	67.83 [13.36]	0.586
YFAS level of FA	0.86 [1.46]	0.38 [1.06]	0.356	0.5 [1.07]	1.14 [1.35]	0.280	0.17 [0.41]	0.67 [1.21]	0.363

EuroQ_tot, European Quality of Life total score; FAS, fibromyalgia assessment status; FIQR_tot, revised fibromyalgia impact questionnaire total score; FSS, fibromyalgia severity scale; DERS, difficulties in emotional regulation scale; YFAS, Yale food addiction scale; FA, food addiction; KDCB, ketogenic diet and carboxytherapy group; KD, ketogenic diet group; CB, carboxytherapy group; T0, baseline; T1, after ten weeks. Standard deviation in square brackets. Statistical significance was attributed as * *p* < 0.05.

## Data Availability

The data presented in this study are available on request from the corresponding author. The data are not publicly available due to privacy.

## Appendix A

In Appendix A, the EQ-5D, FAS, FSS, FIQR, DERS, and YFAS 2.0 questionnaires are reported.

**Euro-Qol 5D** Indicare quali delle seguenti affermazioni descrive meglio il suo stato di salute oggi, segnando con una crocetta (☒) una sola casella di ciascun gruppo											
**Capacità di movimento** Non ho difficoltà nel camminare Ho qualche difficoltà nel camminare Sono costretto/a a letto											☐ ☐ ☐
**Cura della persona** Non ho difficoltà nel prendermi cura di me stesso Ho qualche difficoltà nel lavarmi o vestirmi Non sono in grado di lavarmi o vestirmi											☐ ☐ ☐
**Attività abituali** *(per es. lavoro, studio, lavori domestici, attività familiari o di svago)* Non ho difficoltà nello svolgimento delle attività abituali Ho qualche difficoltà nello svolgimento delle attività abituali Non sono in grado di svolgere le mie attività abituali											☐ ☐ ☐
**Dolore, Fastidio o malessere** Non provo alcun dolore o fastidio Provo dolore o fastidio moderati Provo estremo dolore o fastidio											☐ ☐ ☐
**Ansia o Depressione** Non sono ansioso o depresso Sono moderatamente ansioso o depresso Sono estremamente ansioso o depresso											☐ ☐ ☐
Per aiutarla ad esprimere il suo stato di salute attuale abbiamo disegnato una scala graduate (simile ad un termometro) sulla quale il migliore stato di salute immaginabile è contrassegnato dal numero 100 e il peggiore dallo 0. Vorremmo che indicasse su questa scala quale è, secondo lei, il livello del suo stato di salute oggi, tracciando una linea dal riquadro sottostante fino al punto che corrisponde al suo stato di salute attuale.											Migliore stato di salute immaginabile Peggiore stato di salute immaginabile
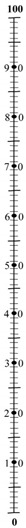
**Fibromyalgia Assessment Status**											
(1) Quale numero da 0 a 10 descrive nel miglioro modo il livello di fatica che hai provato nella scorsa settimana?											
Nessuna stanchezza	1	2	3	4	5	6	7	8	9	10	La peggior stanchezza
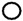
(2) Quanti problemi ha avuto per dormire nella scorsa settimana?											
Nessun problema	1	2	3	4	5	6	7	8	9	10	Numerosi problemi
(3) Si prega di indicare sotto la sensazione di dolore da o a 4 che hai provato nella settimana passata per ciascuna parte del corpo inserendo una X nelle caselle. Si prega di essere sicuri di contrassegnare entrambi i lati destro e sinistro separatamente.											

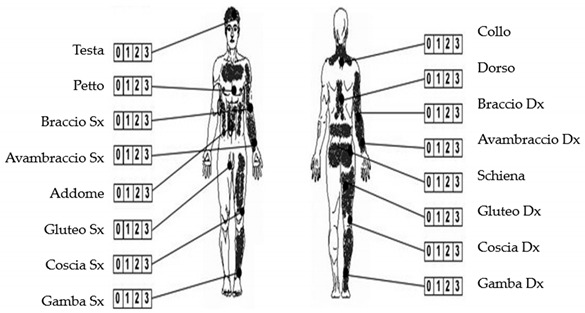

**Fibromyalgia Severity Scale**

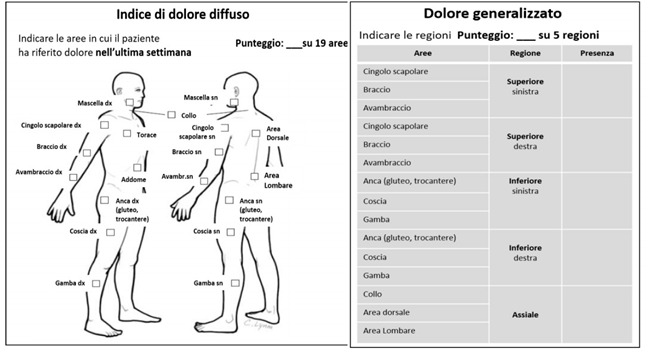

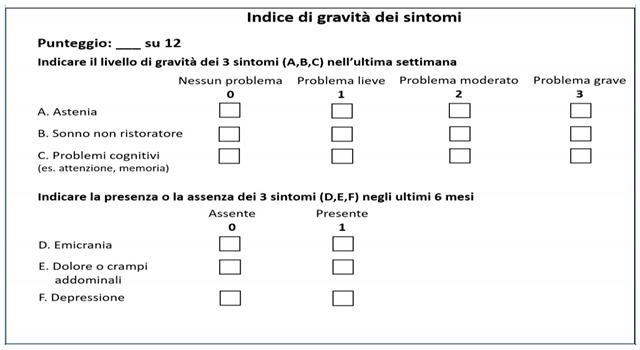

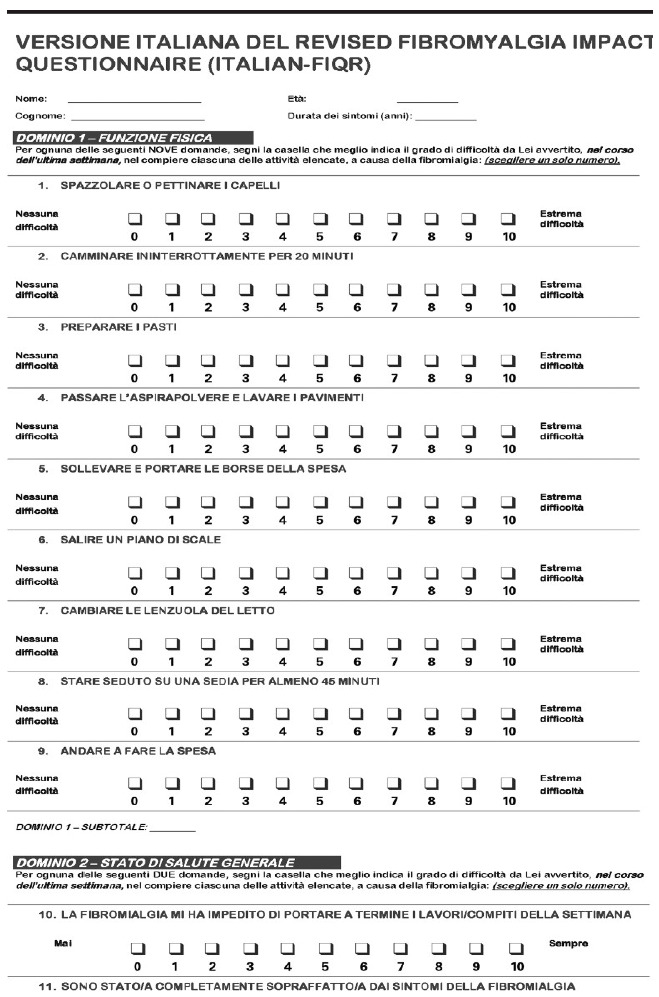

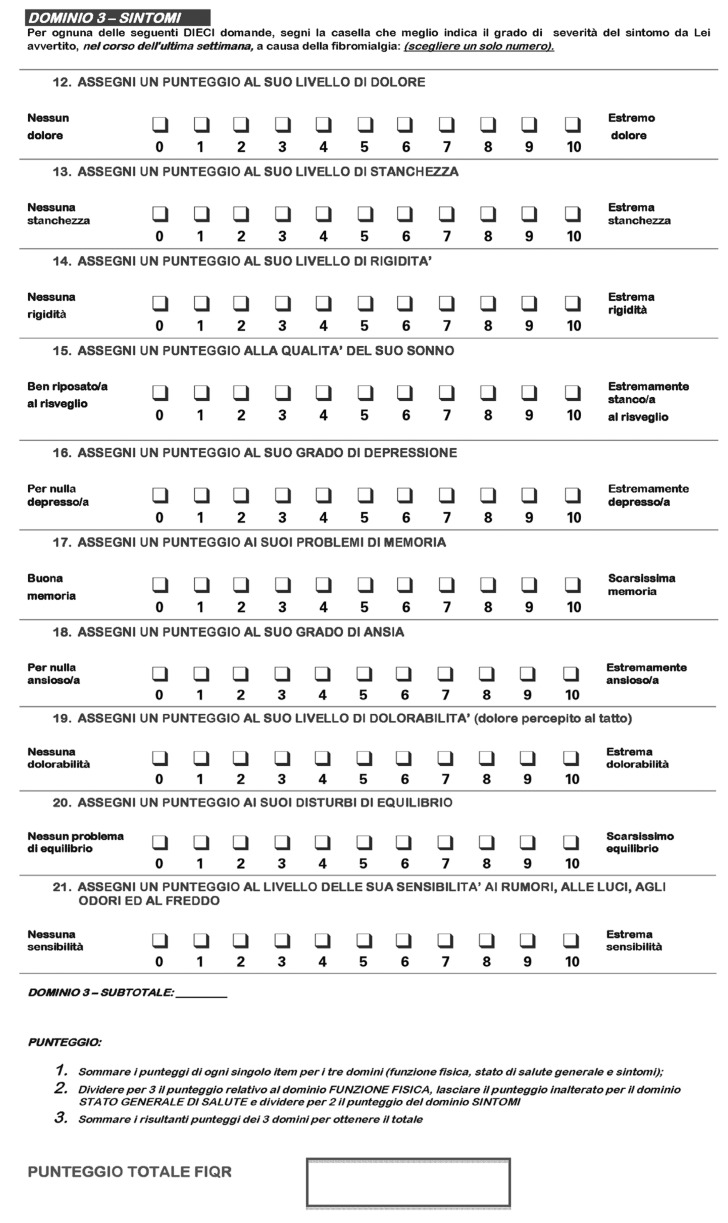

**DERS** Istruzioni Utilizzando la seguente scala di valori, le chiediamo di segnare quanto spesso le seguenti affermazioni possono essere applicate alla sua esperienza, indicando il numero appropiato a fianco di ogni item. 1 Quasi mai (0–10%); 2 A volte (11–35%); 3 Circa la metà delle volte (36–65%); 4 Molte volte (66–90%); 5 Quasi sempre (91–100%).		Quasi sempre	Molte volte	Circa la metà delle volte	A volte	Quasi mai
1	Sono sereno riguardo a ciò che provo	1	2	3	4	5
2	Presto attenzione a come mi sento	1	2	3	4	5
3	Vivo le mie emozioni come travolgenti e fuori dal controllo	5	4	3	2	1
4	Non ho idea di come mi sento	5	4	3	2	1
5	Ho difficoltà a dare un senso a ciò che provo	5	4	3	2	1
6	Presto attenzione alle mie emozioni	1	2	3	4	5
7	So esattamente come mi sento	1	2	3	4	5
8	Mi interessa come mi sento	1	2	3	4	5
9	Sono confuso riguardo a ciò che provo	5	4	3	2	1
10	Quando sono turbato, riconosco le mie emozioni	1	2	3	4	5
11	Quando sono turbato, mi arrabbio con me stesso perché mi sento in quel modo	5	4	3	2	1
12	Quando sono turbato mi imbarazza sentirmi in quel modo	5	4	3	2	1
13	Quando sono turbato, ho delle difficoltà a completare il mio lavoro	5	4	3	2	1
14	Quando sono turbato, perdo il controllo	5	4	3	2	1
15	Quando sono turbato, credo che rimarrò in quello stato per molto tempo	5	4	3	2	1
16	Quando sono turbato, credo che finirò per sentirmi depresso	5	4	3	2	1
17	Quando sono turbato, credo che i miei sentimenti siano validi e importanti	1	2	3	4	5
18	Quando sono turbato, faccio fatica a focalizzarmi su altre cose	5	4	3	2	1
19	Quando sono turbato, mi sento senza controllo	5	4	3	2	1
20	Quando sono turbato, posso comunque finire le cose che devo fare	1	2	3	4	5
21	Quando sono turbato, mi vergogno con me stesso perché mi sento in quel modo	5	4	3	2	1
22	Quando sono turbato, so che alla fine posso trovare un modo per sentirmi meglio	1	2	3	4	5
23	Quando sono turbato, mi sento debole	5	4	3	2	1
24	Quando sono turbato, sento di poter avere ancora il controllo dei miei comportamenti	1	2	3	4	5
25	Quando sono turbato, mi sento in colpa perchè mi sento in quel modo	5	4	3	2	1
26	Quando sono turbato, ho delle difficoltà a concentrarmi	5	4	3	2	1
27	Quando sono turbato, ho delle difficoltà nel controllare i miei comportamenti	5	4	3	2	1
28	Quando sono turbato, credo che non ci sia niente che io possa fare per sentirmi meglio	5	4	3	2	1
29	Quando sono turbato, mi irrito con me stesso perché mi sento in quel modo	5	4	3	2	1
30	Quando sono turbato, inizio a sentirmi molto male con me stesso	5	4	3	2	1
31	Quando sono turbato, credo che crogiolarmi in questa emozione sia l’unica cosa che io possa fare	5	4	3	2	1
32	Quando sono turbato, perdo il controllo sui miei comportamenti	5	4	3	2	1
33	Quando sono turbato, faccio fatica a pensare a qualcosa di diverso	5	4	3	2	1
34	Quando sono turbato, mi prendo del tempo per riflettere su quello che sto provando veramente	1	2	3	4	5
35	Quando sono turbato, mi ci vuole molto tempo per sentirmi meglio	5	4	3	2	1
36	Quando sono turbato, le mie emozioni sono travolgenti	5	4	3	2	1

**I-YFAS 2.0**									
ISTRUZIONI									
Il questionario vuole indagare sulle sue abitudini alimentari nell’ultimo anno. Alcune persone hanno difficoltà a controllare l’assunzione di determinati cibi come: -Dolci quali gelati, cioccolato, ciambelle, biscotti, torte, caramelle -Amidi quali pane bianco, pasta e riso -Snack salati quali patatine, crackers ecc -Cibi grassi quali hamburger, pancetta, pizza, patatine fritte ecc -Bevande gassate quali coca cola, fanta ecc Nelle domande la dizione “certi cibi” si riferisce a qualsiasi tipo di cibo menzionato sopra o comunque a qualsiasi tipo di cibo che ritenga possa essere coinvolto.		Ogni Giorno	4–6 Volte a settimana	2–3 Volte a Settimana	1 Volta a settimana	2–3 Volte al Mese	1 Volta al Mese	Non Mensilemtente	Mai
1	Quando inizio a mangiare “certi cibi” finisco per mangiare molto più di quanto pianificato.	7	6	5	4	3	2	1	0
2	Continuo a mangiare “certi cibi” nonostante non sia più affamato.	7	6	5	4	3	2	1	0
3	Mangio fino a sentirmi male fisicamente.	7	6	5	4	3	2	1	0
4	Non mangiare “certi cibi” o ridurne certi altri è qualcosa che mi preoccupa.	7	6	5	4	3	2	1	0
5	Trascorro tanto tempo sentendomi pieno o affaticato per aver mangiato troppo.	7	6	5	4	3	2	1	0
6	Mi trovo costantemente a mangiare “certi cibi” durante tutto il giorno.	7	6	5	4	3	2	1	0
7	Trovo il modo di procurarmi “certi cibi” quando non sono disponibili. Per esempio, sarei disposto ad allungare il mio percorso di guida verso un negozio per comprarli, anche quando ho altre opzioni disponibili a casa.	7	6	5	4	3	2	1	0
8	Ho mangiato “certi cibi” così spesso o in quantità tale che ho smesso di fare altre cose importanti, come lavorare o passare il tempo con la mia famiglia o con gli amici.	7	6	5	4	3	2	1	0
9	Ho avuto problemi con la mia famiglia o con i miei amici per aver mangiato troppo.	7	6	5	4	3	2	1	0
10	Ho evitato la scuola, il lavoro o attività sociali per paura di mangiare in eccesso lì	7	6	5	4	3	2	1	0
11	Quando ho ridotto o smesso di mangiare “certi cibi” mi sono sentito irritabile, nervoso/a o triste.	7	6	5	4	3	2	1	0
12	Quando ho avuto sintomi fisici, per non aver mangiato “certi cibi”, li ho mangiati per sentirmi meglio.	7	6	5	4	3	2	1	0
13	Quando ho avuto disturbi emotivi, per non aver mangiato “certi cibi”, li ho mangiati per sentirmi meglio.	7	6	5	4	3	2	1	0
14	Quando ho ridotto o smesso di mangiare “certi cibi” ho avuto sintomi fisici. Per esempio ho avuto mal di testa o stanchezza.	7	6	5	4	3	2	1	0
15	Quando ho ridotto o smesso di mangiare “certi cibi” ho avuto gran voglia di mangiarli.	7	6	5	4	3	2	1	0
16	La mia condotta alimentare mi ha causato molto malessere.	7	6	5	4	3	2	1	0
17	Ho avuto problemi significativi nella mia vita a causa del cibo e della alimentazione. Questi problemi possono essere relazionati con la mia routine, il lavoro, la scuola, gli amici, la famiglia o la salute.	7	6	5	4	3	2	1	0
18	Mi sono sentito male per aver mangiato in eccesso, tanto da non fare altre cose importanti. Tra cui lavorare o passare il tempo con la famiglia e gli amici.	7	6	5	4	3	2	1	0
19	Mangiare in eccesso ha interferito nel adempimento dei miei doveri familiari o nel fare i servizi domestici.	7	6	5	4	3	2	1	0
20	Ho evitato il lavoro, la scuola o le attività perché in queste situazioni non potevo mangiare “certi cibi”.	7	6	5	4	3	2	1	0
21	Ho evitato occasioni/incontri sociali perché la gente non avrebbe approvato quanto mangio.	7	6	5	4	3	2	1	0
22	Nonostante sapessi che la mia alimentazione avrebbe causato disturbi emotivi, ho continuato a mangiare “certi cibi”.	7	6	5	4	3	2	1	0
23	Nonostante sapessi che la mia alimentazione mi avrebbe causato problemi fisici, ho continuato a mangiare “certi cibi”	7	6	5	4	3	2	1	0
24	Mangiare la stessa quantità di cibo non mi da più tanta soddisfazione come prima.	7	6	5	4	3	2	1	0
25	Realmente ho voluto ridurre o smettere di mangiare “certi cibi”, però semplicemente non ci sono riuscito.	7	6	5	4	3	2	1	0
26	Avevo bisogno di mangiare ogni volta di più per raggiungere la sensazione che volevo raggiungere mangiando. Ciò includeva, ridurre le emozioni negative (come la tristezza) o aumentare le emozioni positive (come il piacere).	7	6	5	4	3	2	1	0
27	Non ho reso molto a lavoro o a scuola perché stavo mangiando troppo.	7	6	5	4	3	2	1	0
28	Ho continuato a mangiare “certi cibi”, nonostante sapessi che era dannoso per la mia salute. Per esempio, ho continuato a mangiare dolci pur avendo il diabete, o cibi grassi, pur avendo malattie cardiovascolari.	7	6	5	4	3	2	1	0
29	Ho avuto impulsi tanto forti per mangiare “certi cibi” che non potevo pensare ad altro.	7	6	5	4	3	2	1	0
30	Ho avuto desideri tanto intensi per “certi cibi” che sentivo che avrei dovuto mangiarli nell’immediato.	7	6	5	4	3	2	1	0
31	Ho provato a ridurre o smettere di mangiare “certi cibi”, però non ho avuto successo.	7	6	5	4	3	2	1	0
32	Ho provato a ridurre o smettere di mangiare “certi cibi” ed ho fallito.	7	6	5	4	3	2	1	0
33	Sono stato tanto distratto/a mangiando che ho rischiato di ferirmi (ad esempio guidando l’auto, attraversando la strada, maneggiando macchinari).	7	6	5	4	3	2	1	0
34	Sono stato tanto distratto/a pensando al cibo che ho rischiato di ferirmi (ad esempio guidando l’auto, attraversando la strada, maneggiando macchinari).	7	6	5	4	3	2	1	0
35	I miei amici o familiari sono stati molto preoccupati perché ho esagerato con il cibo.	7	6	5	4	3	2	1	0
